# Prevalence of frailty and association with patient centered outcomes: A prospective registry-embedded cohort study from India

**DOI:** 10.1016/j.jcrc.2023.154509

**Published:** 2024-04

**Authors:** Bharath Kumar Tirupakuzhi Vijayaraghavan, Aasiyah Rashan, Lakshmi Ranganathan, Ramesh Venkataraman, Swagata Tripathy, Devachandran Jayakumar, Pratheema Ramachandran, Zubair Umer Mohamed, Sindhu Balakrishnan, Nagarajan Ramakrishnan, Rashan Haniffa, Abi Beane, Neill K.J. Adhikari, Nicolette de Keizer, Nazir Lone, the IRIS collaborative

**Affiliations:** a Department of Critical Care Medicine, Apollo Hospitals, Chennai, India; b The George Institute for Global Health, New Delhi, India; c Network for Improving Critical care Systems and Training, Colombo, Sri Lanka; d Department of Anaesthesia and Critical Care Medicine, All India Institute of Medical Sciences, Bhubaneswar, India; e Department of Critical Care Medicine, Apollo Specialty Hospital, Chennai, India; f Department of Critical Care Medicine, Dr. Kamakshi Memorial Hospital, Chennai, India; g Department of Anaesthesia and Critical Care Medicine, Amrita Institute of Medical Sciences, Amrita Vishwa Vidyapeetham, Kochi, India; h Mahidol Oxford Tropical Medicine Research Unit, Bangkok, Thailand; i Centre for Inflammation Research, University of Edinburgh, United Kingdom; j Interdepartmental Division of Critical Care Medicine, University of Toronto, Canada; k Department of Critical Care Medicine, Sunnybrook Health Sciences Centre, Toronto, Canada; l Department of Medical Informatics, Amsterdam Public Health Research Institute, Amsterdam UMC, Amsterdam, the Netherlands; m Usher Institute, University of Edinburgh, Edinburgh, United Kingdom; n University College, London

**Keywords:** Frailty, Intensive care units, Registries, Global Health, Developing countries

## Abstract

**Purpose:**

We aimed to study the prevalence of frailty, evaluate risk factors, and understand impact on outcomes in India.

**Methods:**

This was a prospective registry-embedded cohort study across 7 intensive care units (ICUs) and included adult patients anticipated to stay for at least 48 h. Primary exposure was frailty, as defined by a score ≥ 5 on the Clinical Frailty Scale and primary outcome was ICU mortality. Secondary outcomes included in-hospital mortality and resource utilization. We used generalized linear models to evaluate risk factors and model association between frailty and outcomes.

**Results:**

838 patients were included, with median (IQR) age 57 (42,68) yrs.; 64.8% were male. Prevalence of frailty was 19.8%. Charlson comorbidity index (OR:1.73 (95%CI:1.39,2.15)), Subjective Global Assessment categories mild/moderate malnourishment (OR:1.90 (95%CI:1.29, 2.80)) and severe malnourishment (OR:4.76 (95% CI:2.10,10.77)) were associated with frailty. Frailty was associated with higher odds of ICU mortality (adjusted OR:2.04 (95% CI:1.25,3.33)), hospital mortality (adjusted OR:2.36 (95%CI:1.45,3.84)), development of stage2/3 AKI (unadjusted OR:2.35 (95%CI:1.60, 3.43)), receipt of non-invasive ventilation (unadjusted OR:2.68 (95%CI:1.77, 4.03)), receipt of vasopressors (unadjusted OR:1.47 (95%CI:1.04, 2.07)), and receipt of kidney replacement therapy (unadjusted OR:3.15 (95%CI:1.90, 5.17)).

**Conclusions:**

Frailty is common among critically ill patients in India and is associated with worse outcomes.

Study registration: CTRI/2021/02/031503.

## Highlights

• There is limited information on frailty and its impact on outcomes from India and other lower-middle income countries.
• In this registry-embedded cohort study that enrolled 838 patients from 4 ICUs in India, prevalence of frailty was 20%.
• Frailty was associated with higher ICU mortality, hospital mortality and receipt of organ support.

## 1 Introduction

Frailty is a complex syndrome characterized by a loss of reserve in several physiological domains (energy, cognitive ability, physical capacity) that renders the individual susceptible to adverse outcomes in the face of illness. [1,2] Frailty was originally described in the geriatric population and demonstrated to have associations with mortality, risk of hospitalization, falls and disability. [[3], [4], [5], [6]] It is unsurprising that a significant proportion of patients admitted to Intensive Care Units (ICUs) share these traits, with pre-existing functional and cognitive limitations and multimorbidity, and are vulnerable to adverse outcomes. [7,8]

Studies from high-income countries have demonstrated associations between pre-admission frailty and poor outcomes, including mortality and poor health-related quality of life. [[9], [10], [11], [12]]. There is limited data on the prevalence of frailty and the associated risk factors among critically ill patients in lower-middle income countries (LMICs). Data from at least one study in India identified the presence of frailty in 28% of general community dwellers. [13] Differences in per capita income, nutritional status, burden of infectious diseases, access to quality healthcare, and social safety nets among other factors may contribute to frailty in unique ways in India and other LMICs. Therefore, we aimed to study the prevalence of frailty on admission to the intensive care unit (ICU) among critically ill patients in India, evaluate risk factors for frailty, and understand the impact of frailty on patient-centred outcomes. Our prespecified hypotheses were that frailty would be common among critically ill patients in India and would be associated with worse outcomes, including higher ICU mortality.

## 2 Methods

### 2.1 Study design and setting

This was a prospective registry-embedded cohort study conducted across 7 ICUs (from 4 hospitals) affiliated with the Indian Registry of IntenSive care (IRIS) [14] from 11th June 2021 to 26th December 2021. The participating hospitals represented a combination of private and public healthcare organizations and were chosen in order to provide a reasonable degree of representativeness. Details of the participating sites with the number of patients enrolled from each site are provided in Supplementary Table 1.

IRIS, a cloud-based registry of ICUs in India, was set up in 2019 to enable the evaluation of case-mix and outcomes in Indian ICUs, facilitate audit and quality improvement and embed observational research and clinical trials. Additional details have been published previously. [[14], [15], [16]] This study was approved by the Institutional Ethics Committee at the coordinating centre (AMH-023/08–19) and at all participating sites; informed consent was mandated at 2 sites. The study was registered on Clinical Trials Registry of India (CTRI/2021/02/031503) and is reported as per the Strengthening the Reporting of Observational Studies in Epidemiology (STROBE) guidance. [17]

### 2.2 Participants, exposure and outcome variables

All adult (≥18 years) critically ill patients admitted to participating ICUs for the first time during the current hospitalization, and with an anticipated ICU stay of ≥48 h, were included. The key exposure of interest was frailty, represented as a binary variable, as defined by a score on the Clinical Frailty Scale (CFS) of at least 5. [1] CFS is a 9-point scoring system that provides a multi-dimensional estimate of a patient's baseline frailty status. It extends from Level 1 (very fit) to Level 9 (terminally ill). This baseline assessment was defined by the patient's status in the 4 weeks prior to admission (see suppl. Figure1) by interviewing the patient (where possible) or the next of kin. A secondary exposure, based on results of published work [18], was ‘patient- or family-reported decline in functional status’ in the one year prior to ICU admission, determined as a single ‘yes’ or ‘no’ response at admission.

The primary outcome of interest was ICU mortality, censored at day 28. Secondary outcomes were in-hospital mortality, development of stage 2/3 Acute Kidney Injury (AKI) as per the Kidney Disease Improving Global Outcomes (KDIGO) definitions [19], and measures of resource utilization including receipt of invasive or non-invasive ventilation during the index ICU admission, receipt of vasopressors, receipt of kidney replacement therapy during the index ICU admission, and length of ICU and hospital stay. All outcomes were censored at day 28.

For the evaluation of risk factors for frailty, data were collected on age, sex, comorbidity burden (using the Charlson comorbidity index [20], socioeconomic status (SES) using the modified Kuppuswamy scale [21] and nutritional status at ICU admission using the Subjective Global Assessment (SGA) tool. [22] The modified Kuppuswamy scale incorporates information on education, occupation and income (total score ranging from 3 to 29) to classify individuals into 5 groups, ‘upper class’ ‘upper middle class’, ‘lower middle class’ ‘upper lower’ and ‘lower’ socioeconomic class. [21] We also collected information on reasons for admission and severity of illness at admission using the Acute Physiology and Chronic Health Evaluation II (APACHE II) model. [23]

### 2.3 Data sources, training, measurement and monitoring

Information on demographics, admission diagnoses, severity and clinical outcomes are routinely available from IRIS at the participating hospitals. Additional variables relating to the assessment of frailty, and specific outcomes such as ‘development of stage 2/3 AKI’ were added to the registry at the beginning of the study. Information on SES was obtained by interviewing patients (where feasible) or family members using the modified Kuppuswamy scale. [21] Baseline nutritional status was assessed by trained nutrition specialists or equivalent staff at all participating hospitals using the SGA tool. [22] Information on SES and SGA was then entered onto the registry by site research staff.

For the assessment of frailty with CFS, the study principal investigator (PI) trained site research staff. Following the training session, site investigators and the research associate piloted the CFS tool by independently assessing frailty as a pilot on 5 patients. Similar training was provided for the assessment of the SES. Throughout the study duration, the treating team was blinded to the frailty assessments.

The study was centrally coordinated by the PI and a Project Coordinator. In addition, a study steering committee comprising the PI and all the site investigators met periodically to review progress and address issues as needed.

### 2.4 Sample size

Based on data from high-income countries, the prevalence of frailty in ICU populations ranges from 25 to 30%. [7,12] There are no data on the prevalence of frailty among critically ill patients in India. One study of community-dwelling adults documented a prevalence of 28%. [13] In feasibility work from our ICU (unpublished data), 8% of patients were frail. For the purposes of sample size calculation, we assumed a prevalence of frailty of 10%. Baseline ICU mortality among registry ICUs is 20%. We assumed that the risk of mortality was 35% among frail patients. With a significance level of 5% and type 2 error at 10%, we estimated a sample size of 720 patients. Assuming a 10% loss to follow-up, we inflated our sample size to 800 patients.

### 2.5 Statistical analysis

Categorical variables were reported as counts and percentages and continuous variables were reported as median (interquartile range (IQR)). A Chi-squared test was used to compare categorical variables and a Mann-Whitney test was used to compare continuous non-normally distributed variables.

Missing data for the SGA score and for components of the SES score were imputed using multiple imputation by chained equations, assuming that data were missing at random. Twenty datasets were imputed using predictive mean matching. The imputation dataset consisted of age, sex, Charlson Comorbidity Index, Acute Physiology and Chronic Health Evaluation II (APACHE II) score, ICU mortality, SGA score, the components of the SES and CFS. The imputed datasets were used for all subsequent regression analyses and the results were pooled using Rubin's rules. [24,25]

**Fig. 1:**
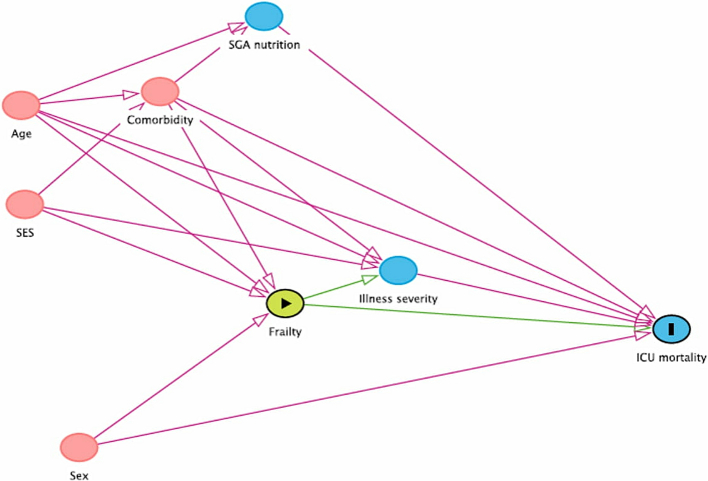
Directed acyclic graph of potential causal pathways for the effect of frailty on ICU mortality.

The primary analysis fitted logistic regression models evaluating the association between frailty and ICU mortality. The adjustment variables were age, sex, APACHE II score, Charlson comorbidity index, SGA score and SES score, chosen on the basis of a prespecified causal framework (Fig. 1). Since the assumptions of linearity were violated for age, APACHE II score, and Charlson comorbidity index, we fitted these terms as fractional polynomials. The regression models were repeated after excluding APACHE II score from the covariate set, since APACHE II score could be a potential mediator between frailty and mortality. We also fitted a logistic regression model for hospital mortality, with the same adjustment variables. Comparisons of other outcomes between frail and non-frail patients are all unadjusted.

To evaluate risk factors for frailty, a multivariable logistic regression model was fitted with frailty at admission as the dependent variable and age, sex, Charlson comorbidity index, SGA, and SES as independent variables. These variables were chosen based on prior literature and hypothesized risk factors in the context of a LMIC setting.

### 2.6 Sensitivity analyses

For the primary analysis, patients who left the ICU against medical advice (LAMA) were considered to have been discharged alive. Because these patients may be more likely to die [26], we conducted a best-worst and worst-best sensitivity analysis. The best-worst analysis considered non-frail patients with LAMA status to be alive, while frail patients were considered dead. The worst-best analysis considered non-frail patients with LAMA status to be dead while frail patients were considered alive.

The primary analysis was repeated using ‘patient or family reported decline in functional status’ as the secondary exposure variable and results are separately reported.

## 3 Results

From 11th June 2021 to 26th December 2021, we screened 2283 patients and enrolled 838 patients (suppl. Figure2). The median age of the cohort was 57 years (IQR 42,68) (supplementary fig. 4) and 64.8% (*n* = 543) of patients were male (Table 1). The median APACHE II score was 12 (IQR 7,17) and the median Charlson comorbidity index was 2 (IQR 0,3). The prevalence of frailty at admission was 19.8% (*n* = 166) (95%CI 17.3%, 22.6%). Compared with non-frail patients, frail patients were older, were more severely ill on admission, were more likely to have a medical reason for admission, were less likely to be well-nourished, and had a greater burden of comorbidity (all *p* < 0.001). Frail patients or their families were twice as likely to report a perceived decline in functional status in the one year prior to admission compared with non-frail patients or families (78.3% vs 37.5%, p < 0.001). A higher proportion of frail patients belonged to the ‘Upper Middle’ SES category and a higher proportion of non-frail patients belonged in the ‘Lower middle’ and ‘Upper lower’ categories (*p* = 0.02).

**Table 1:** Baseline characteristics of patients.

Variable	Total (*N* = 838)	Frail (*N* = 166)	Not frail (*N* = 672)	p
Age, Median (IQR)	57 (42,68)	68 (52,76)	55 (40,65)	**<0.001**
Male, N(%)	543 (64.8)	110 (66.3)	433 (64.4)	0.72
Weight, Median (IQR)	62 (55,70)	64 (55,70)	62 (55,70)	0.76
APACHE II, Median (IQR)	12(7,17)	15(10,21)	11(7,16)	**<0.001**
Charlson score, Median (IQR)	2 (0,3)	3 (1,4)	1 (0,3)	**<0.001**
Patient or family reported decline in functional status, N(%)				**<0.001**
No	434 (51.8)	34 (20.5)	400 (59.5)	
Yes	382 (45.6)	130 (78.3)	252 (37.5)	
Missing	22 (2.6)	2 (1.2)	20 (3.0)	
Type of admission, N(%)				**<0.001**
Medical	575 (68.6)	142 (85.5)	433 (64.4)	
Surgical	263 (31.4)	24 (14.5)	239 (35.6)	
Socioeconomic status⁎, N(%)				0.02
Upper (I)	35 (4.2)	11 (6.6)	24 (3.6)	
Upper Middle (II)	247 (29.5)	54 (32.5)	193 (28.7)	
Lower Middle (III)	195 (23.3)	36 (21.7)	159 (23.7)	
Upper Lower (IV)	253 (30.2)	38 (22.9)	215 (32.0)	
Lower (V)	6 (0.7)	0 (0.0)	6 (0.9)	
Missing	102 (12.2)	27 (16.3)	75 (11.2)	
Subjective Global Assessment#, N(%)				**<0.001**
A - Well-nourished, Normal	492 (58.7)	75 (45.2)	417 (62.1)	
B- Mildly/moderately malnourished, Some progressive nutritional loss	308 (36.8)	75 (45.2)	233 (34.7)	
C - Severely malnourished, Evidence of wasting and progressive symptoms	30 (3.6)	15 (9.0)	15 (2.2)	
Missing	8 (1.0)	1 (0.6)	7 (1.0)	
Reasons for ICU admission, N (%)^				
Cardiovascular	125(14.9)	35(21.1)	90(13.4)	
Neurological	173(20.6)	32(19.3)	141(21.0)	
Sepsis	124(14.8)	39(23.5)	85(12.6)	
Trauma	52(6.2)	3(1.8)	49(7.3)	
Others	471(56.2)	98(59.0)	373(55.5)	

⁎ Socioeconomic status is reported using the Modified Kuppuswamy Scale [21].

# Nutritional status at admission is reported using the validated Subjective Global Assessment tool [22].

^ Patients can have more than one reason for ICU admission.

Table 2 lists the associations between frailty and primary and secondary outcomes. Thirty-six patients in the frail group (21.7%) and 81 patients in the non-frail group (12.1%) died in the ICU. An additional 26 patients (3.1%) (18 in the non-frail group and 8 in the frail group) were discharged against medical advice. Frailty was associated with a higher odds of ICU mortality (adjusted OR:2.04 (95% CI:1.25,3.33), *p* = 0.005). (Table 2) This association remained in regression models excluding APACHE II (adjusted OR:2.27, (95%CI 1.40,3.68), *p* = 0.001). In a sensitivity analysis that included patients that LAMA, this association with higher mortality was also preserved. (suppl.Table 2).

**Table 2:** Outcomes.

Outcome	Frail (*N* = 171)	Not frail (*N* = 668)	Unadjusted OR	95% CI	Adjusted OR	95% CI	p^
ICU mortality, N(%)	36 (21.7)	81 (12.1)	2.02	1.30, 3.11	2.04	1.25, 3.33	0.005
ICU mortality (without APACHE 2)					2.27	1.40, 3.68	0.001
Hospital mortality, N(%)	39(23.5)	81 (12.1)	2.22	1.44, 3.39	2.36	1.45,3.84	0.001
Hospital mortality (without APACHE 2)					2.51	1.55, 4.05	<0.001
Receipt of invasive ventilation⁎, N(%)	88(53.0)	338 (50.3)	1.11	0.79, 1.57			NA
Receipt of non-invasive ventilation⁎, N(%)	46 (27.7)	84 (12.5)	2.68	1.77, 4.03			NA
Receipt of KRT⁎, #, N(%)	30 (18.1)	44 (6.5)	3.15	1.90, 5.17			NA
Receipt of vasopressors⁎, N(%)	97 (58.4)	329 (49.0)	1.47	1.04, 2.07			NA
Development of stage 2 or 3 AKI⁎, &, N(%)	55 (33.1)	117(17.4)	2.35	1.60, 3.43			NA

⁎ Until Day 28 or ICU discharge or death whichever was earlier. These analyses are all unadjusted.

# KRT: Kidney Replacement Therapy.

& As per Kidney Diseases Improving Global Outcomes(KDIGO) criteria.

^ *p* values are for the adjusted analyses.

Frailty was similarly associated with a higher odds of hospital mortality (adjusted OR:2.36 (95%CI:1.45,3.84), p = 0.001), receipt of non-invasive ventilation (unadjusted OR: 2.68 (95%CI:1.77,4.03)), receipt of kidney replacement therapy (unadjusted OR: 3.15 (95%CI:1.90,5.17)), receipt of vasopressors (unadjusted OR: 1.47 (95%CI:1.04,2.07)), and development of stage2/3 AKI (unadjusted OR:2.35 (95%CI:1.60,3.43)). Frail patients also had longer ICU stay (median of 6 days vs. 4 days among non-frail patients; *p* < 0.001) (suppl.Table 3). However, our analysis did not demonstrate an association between frailty and receipt of invasive ventilation (unadjusted OR:1.11 (95%CI:0.79,1.57)).

**Table 3:** Factors associated with frailty at admission.

Variable	Odds Ratio	Lower 95% CI	Upper 95% CI	p
Age⁎	0.98	0.96	1.00	0.12
Sex- Male⁎	0.98	0.66	1.43	0.90
Charlson score⁎	1.73	1.39	2.15	**<0.001**
SGA reference category Well-nourished, Normal				
SGA Mildly/moderately malnourished	1.90	1.29	2.80	**0.001**
SGA Severely malnourished	4.76	2.10	10.77	**<0.001**
SES reference category Upper (I)				
SES Upper Middle (II)	0.60	0.26	1.35	0.21
SES Lower Middle (III)	0.60	0.26	1.35	0.21
SES Upper Lower or Lower (IV/V)	0.40	0.17	0.92	0.03

⁎ Odds ratios are reported for per year increase in age and per point increase in Charlson comorbidity index. For sex, female is the reference.

Risk factors associated with higher frailty at ICU admission (Table 3) included Charlson comorbidity index (OR:1.73 (95%CI:1.39,2.15)) and nutritional status on admission (SGA categories mild/moderate malnourishment vs well nourished (OR:1.90 (95%CI:1.29,2.80)) and severe malnourishment (OR:4.76 (95% CI:2.10,10.77)). In addition, SES, specifically belonging to Upper Lower and Lower categories, was associated with lower frailty on admission compared with the Upper SES category (suppl.Figure3). Age and sex were not associated with frailty status on admission.

When considering patient or family reported decline in functional status in the one year prior to admission as the exposure variable (suppl.Tables 4 and 5), we did not demonstrate an association with ICU mortality (unadjusted OR: 1.02 (95%CI:0.65,1.60)).

## 4 Discussion

In this prospective registry-embedded observational study, the prevalence of frailty at ICU admission was 20%. Frailty was associated with a higher odds of ICU mortality, hospital mortality, development of stage2/3 AKI and receipt of non-invasive ventilation, kidney replacement therapy and vasopressors up to day 28. There was no association between frailty and receipt of invasive ventilation. Charlson comorbidity index and malnourishment at admission were risk factors for frailty in our cohort.

Our results are broadly similar to prior work in this field. In a single-centre study from India, the prevalence of frailty at ICU admission was 38.6% and was associated with higher mortality. [27] In two recent systematic reviews [28,29], that included observational studies from high-income and upper-middle income countries, the pooled prevalence of frailty ranged from 30 to 37%. Frailty was associated with higher risk of hospital, short-term and long-term mortality. The pooled results did not demonstrate an association between frailty and receipt of mechanical ventilation. Two of the included studies reported on health-related quality of life with frail patients having a reduced quality of life at 1 year post discharge. [28,29].

The lower prevalence of frailty noted in our cohort as compared to the single-centre study and pooled prevalence from the systematic reviews could be related to differences in the populations studied (the single-centre study from India only included patients older than 50 years), triage decisions based on baseline frailty at the level of the emergency room, or the in-patient wards due to perceived lower benefits of admitting these patients to the ICU.

The finding of a lack of an association between frailty and receipt of invasive ventilation both, in our study and in earlier published meta-analyses, would appear counter-intuitive; particularly so, as receipt of other organ support strategies appeared to be higher for frail patients in our study. Potential explanations include clinical decisions around limiting life support based on frailty or the consequence of residual confounding. Our analyses of ICU and hospital mortality were adjusted for potential confounders, but not those for other outcomes. Given the possibility of false positive findings due to multiple testing, we restricted adjusted analyses to the two outcomes of ICU and hospital mortality.

Specific to India, frailty has been assessed in different contexts; in patients with Chronic Kidney Disease (CKD) [30], in community-dwelling older adults [31], and in rural populations [32], and has been associated with worse outcomes. [33] Conventional determinants of frailty are age, sex, absence of social support, polypharmacy, multiple comorbidities, higher body mass index, and presence of psychological factors among others. [34,35] In our context, we hypothesized that additional factors such as SES and nutrition would have an impact on frailty. Our analysis confirmed the influence of malnutrition, but not of SES on admission frailty. Our results suggest a protective effect of lower SES on the risk on frailty, which we hypothesize is related to a potential collider bias where both frailty and SES act via financial status to influence the decision to admit to the ICU. It is also possible that patients from lower SES strata have greater resilience and are less likely to report frailty.

Previous work [18] evaluated the association between ‘patient or family reported decline in functional status’ and survival at hospital discharge and at 1 year following discharge, demonstrating significant associations between patient or family reported decline and ICU, hospital and 1 year mortality. In contrast, in our study, despite a higher patient or family reported decline in functional status (45.6% compared to 40.4% in the previous analysis), we did not demonstrate an association with hospital mortality or ICU mortality. This finding could potentially be attributable to differences in patient profile or illness severity (hospital mortality in our study was 14.3% as compared to 33.8% in the previous study). It is also possible that this ‘self or family reported decline in functional status’ is addressing a different construct. While the appeal of a single question for the assessment of baseline functional status or to serve as a surrogate for frailty is obvious, further research is needed to understand the usefulness of such assessments.

Given the increase in volume of recent research exploring associations of frailty with clinical outcomes among critically ill patients, Shah and colleagues [36], raise important questions on the usefulness of such research. They argue that studies that describes frailty prevalence or associations with outcomes are unlikely to be useful unless they directly improve the quality of information provided to patients and families as they navigate treatment decisions, or it changes how we deliver care. While we acknowledge their broad concerns, understanding the burden and epidemiology of frailty from different contexts is an essential first step. Specific to LMICs and other resource limited settings, assessment of frailty may help in triage and in informing patient and family decisions around therapeutic choices and goals of care. In terms of improving clinical outcomes, potential target areas for interventions include nutrition, early rehabilitation, and strategies focused on premorbid health status optimisation among others. Presumably, any potential strategy would likely be a complex intervention that transcends numerous domains, given the multidomain nature of frailty itself. In addition, frailty could lend itself as a ‘biomarker’ for critical care trials that employ prognostic enrichment. [37,38] Beyond hospital-based interventions, improving social determinants of health (e.g., clean water, sanitation, access to quality health care etc.) at the national and international level are needed for improving outcomes for these patients, especially in India and other LMICs.

## 5 Strengths and limitations

Our study has important strengths. Ours is the largest study of frailty among critically ill patients from a LMIC setting and the study was conducted at a mix of public and private hospitals as well as academic and non-academic centres, thereby improving the external validity of our findings. We embedded the study in a cloud-based registry to reduce data collection burden and improve data quality. We specifically evaluated risk factors that are contextually relevant, and carefully considered causal pathways using a directed acyclic graph for our analysis.

Important limitations include the lack of follow-up for longer-term outcomes beyond hospital discharge. We assessed frailty once patients were admitted to the intensive care unit and as such, estimates of prevalence do not reflect any triage decisions made at the emergency room level or at the in-patient ward level regarding transfer or admission to the ICU. Finally, we used the Clinical Frailty Scale in our study. CFS is validated, has good inter-rater reliability, [39], is easy-to-apply at the bedside, and is the mostly commonly used tool in studies of critically ill patients. [40] However, the tool has limitations including a lack of robustness in specific contexts such as in patients with dementia, autism and cerebral palsy. [41,42]

## 6 Conclusions

Frailty is common among critically ill patients in India and is associated with worse clinical outcomes. Future research must focus on strategies for improving outcomes for frail ICU patients.

## Funding

This research was funded in part, by the 10.13039/100010269Wellcome Trust [Grant number WT215522/Z/19/Z]. For the purpose of open access, the author has applied a CC BY public copyright licence to any Author Accepted Manuscript version arising from this submission. The funder had no role in the design, conduct, analysis or the decision to submit for publication.

## Data availability and sharing

The IRIS collaboration supports and welcome data sharing. Data will be made available to researchers who provide a detailed and methodologically sound proposal with specific aims that are clearly outlined. Data sharing will be for the purposes of medical research and under the auspices of the consent under which the data were originally gathered.

To gain access, researchers will need to sign a data sharing and access agreement and will need to confirm that data will only be used for the agreed upon purpose for which data access was granted. Researchers can contact the corresponding author through electronic mail (bharath@icuconsultants.com) for such access.

## CRediT authorship contribution statement

**Bharath Kumar Tirupakuzhi Vijayaraghavan:** Conceptualization, Investigation, Methodology, Project administration, Visualization, Writing – original draft, Writing – review & editing. **Aasiyah Rashan:** Data curation, Formal analysis, Visualization, Writing – review & editing. **Lakshmi Ranganathan:** Project administration, Resources, Writing – review & editing. **Ramesh Venkataraman:** Investigation, Writing – review & editing. **Swagata Tripathy:** Investigation, Writing – review & editing. **Devachandran Jayakumar:** Investigation, Writing – review & editing. **Pratheema Ramachandran:** Investigation, Writing – review & editing. **Zubair Umer Mohamed:** Investigation, Writing – review & editing. **Sindhu Balakrishnan:** Investigation, Writing – review & editing. **Nagarajan Ramakrishnan:** Investigation, Writing – review & editing. **Rashan Haniffa:** Conceptualization, Methodology, Supervision, Writing – review & editing. **Abi Beane:** Conceptualization, Methodology, Supervision, Writing – review & editing. **Neill K.J. Adhikari:** Conceptualization, Methodology, Supervision, Writing – review & editing. **Nicolette de Keizer:** Conceptualization, Methodology, Supervision, Writing – review & editing. **Nazir Lone:** Conceptualization, Methodology, Supervision, Writing – review & editing.

## Declaration of Competing Interest

Nicolette de Keizer: None directly related to this work. Her department receives an ongoing funding by the National Intensive Care Evaluation registry, Netherlands for her work.

Abi Beane: Receives part salary support from 10.13039/100010269Wellcome Trust, United Kingdom.

## Appendix A Supplementary data

Supplementary material 1: STROBE checklist Image 1

Supplementary material 2: Additional Supplementary Material and supporting information Image 2
