# Supplementary material for: Prevalence of frailty and association with patient centered outcomes: A prospective registry-embedded cohort study from India
Source: J Crit Care. 2024 Apr;80:None. doi: 10.1016/j.jcrc.2023.154509 (PMC10830405; doi:10.1016/j.jcrc.2023.154509)
Supplement: Supplementary file 2 — Supplementary material 2: Additional Supplementary Material and supporting information [file mmc2.docx]

**Supplementary Materials**

**Supplementary Table 1: Sites and number of patients enrolled per site**

| **Site** | **Type of Hospital** | **Number of patients enrolled** |
| --- | --- | --- |
| Apollo Main Hospital, Chennai (Coordinating centre), India | Private teaching hospital | 236 |
| Apollo Specialty Hospital, Chennai, India | Private non-teaching hospital | 175 |
| All India Institute for Medical Sciences, Bhubaneswar, India | Public/University teaching hospital | 272 |
| Amrita Institute of Medical Sciences, Kochi, India | Private/University teaching hospital | 155 |
| **Total** |  | 838 |

**Supplementary Table 2: Sensitivity analyses of Best-Worst and Worst-Best assumptions**

| **Variable** | **Total (N=838)** | **Frail (N=166)** | **Not frail (N=672)** | **Unadjusted OR (95%CI)** | **p** |
| --- | --- | --- | --- | --- | --- |
| LAMA N(%)^#^ | 26 (3.1) | 8 (4.8) | 18 (2.7) |  | 0.003 |
| ICU mortality Best-Worst N(%)^*^ | 125 (14.9) | 44 (26.5) | 81 (12.1) | 2.63(1.73,2.97) | <0.001 |
| ICU mortality Worst-Best N(%)^*^ | 135 (16.1) | 36 (21.7) | 99 (14.7) | 1.60(1.04,2.44) | 0.03 |

**^#^Left Against Medical Advice**

***For Best-Worst, everyone that was non-frail and LAMA was assumed to be alive and everyone that was frail and went LAMA was assumed to be dead. For Worst-Best, the assumptions were reversed.**

**Supplementary Table 3: Additional outcomes**

| ICU length of stay (days) Median (IQR) | 4 (3 - 8) | 6 (3 - 9) | 4 (2 - 7) |  | <0.001 |
| --- | --- | --- | --- | --- | --- |
| Length of stay (Dead) Median (IQR) | 7 (4 - 13) | 7 (5 - 11) | 7 (4 - 15) |  | 0.50 |
| Length of stay (Alive) Median (IQR) | 4 (2 - 7) | 5 (3 - 8.8) | 4 (2 - 7) |  | <0.001 |
| Days alive and off ICU Median (IQR)* | 23 (17 - 25) | 21 (7.5 - 24) | 24 (18 - 25) |  | <0.001 |

**Supplementary Table 4: Outcomes with respect to patient or family reported decline in functional status**

| **Variable** | **Total (N=838)** | **Yes (N=382)** | **No (N=434)** | **Missing (N=22)** | **Unadjusted**  **OR (95%CI)** | **p** |
| --- | --- | --- | --- | --- | --- | --- |
| ICU mortality N(%) | 117 (14) | 59 (15.4) | 53 (12.2) | 5 (22.7) | 1.31(0.88,1.96) | 0.20 |
| Hospital mortality N(%) | 120 (14.3) | 62 (16.2) | 55 (12.7) | 3 (13.6) | 1.34(0.90,1.98) | 0.35 |
| Received invasive ventilation N(%) | 426 (50.8) | 179 (46.9) | 237 (54.6) | 10 (45.5) | 0.73(0.56,0.97) | 0.08 |
| Received non-invasive ventilation N(%) | 130 (15.5) | 77 (20.2) | 52 (12) | 1 (4.5) | 1.85(1.27,2.73) | <0.001 |
| Received kidney replacement therapy N(%) | 74 (8.8) | 46 (12) | 27 (6.2) | 1 (4.5) | 2.06(1.26,3.43) | 0.01 |
| Received vasopressors N(%) | 426 (50.8) | 196 (51.3) | 222 (51.2) | 8 (36.4) | 1.01(0.76,1.33) | 0.39 |
| Development of stage 2 or 3 AKI N(%) | 172 (20.5) | 86 (22.5) | 85 (19.6) | 1 (4.5) | 1.19(0.85,1.67) | 0.10 |
| ICU length of stay (days) Median (IQR) | 4(3,8) | 5(,8) | 4(3,7) | 3(2,7,5) | NA | 0.04 |
| Length of stay (Dead) Median (IQR) | 7 (4 - 13) | 7 (4 - 13) | 8 (5 - 14) | 8 (5 - 9) | NA | 0.76 |
| Length of stay (Alive) Median (IQR) | 4 (2 - 7) | 5 (3 - 8) | 4 (2 - 6) | 3 (2 - 4) | NA | 0.01 |
| Days alive and off ICU Median (IQR) | 23 (17 - 25) | 23 (15.2 - 25) | 24 (19 - 25) | 24.5 (9.5 - 25.8) | NA | 0.02 |

**Supplementary Table 5: Multivariable logistic regression for functional status and ICU mortality**

| **Variable** | **Odds ratio** | **Lower 95% CI** | **Upper 95% CI** | **p** |
| --- | --- | --- | --- | --- |
| Declined functional status-Yes | 1.02 | 0.65 | 1.60 | 0.93 |
| Age | 1.01 | 0.99 | 1.04 | 0.31 |
| Gender - Male | 1.31 | 0.85 | 2.03 | 0.22 |
| APACHE II score | 1.04 | 1.01 | 1.07 | <0.001 |
| Charlson comorbidity score | 0.98 | 0.76 | 1.27 | 0.90 |
| SGA reference category Well-nourished, Normal |  |  |  |  |
| SGA  Mildly/moderately malnourished,  Some progressive nutritional loss | 1.28 | 0.82 | 1.99 | 0.28 |
| SGA - Severely malnourished, Evidence of wasting and progressive symptoms | 0.86 | 0.28 | 2.67 | 0.80 |
| SES reference category Upper (I) |  |  |  |  |
| SES Upper Middle (II) | 3.10 | 0.43 | 22.30 | 0.26 |
| SES Lower Middle (III) | 3.40 | 0.46 | 25.01 | 0.23 |
| SES Upper Lower/Lower (IV) | 4.80 | 0.66 | 34.82 | 0.12 |

**Supplementary Table 6: Components of the SES score**

| **Variable** | **Total (N=838)** | **Frail (N=166)** | **Not frail (N=672)** | **p** |
| --- | --- | --- | --- | --- |
| **Occupation** N(%) |  |  |  | 0.001 |
| Clerks | 82 (9.8) | 8 (4.8) | 74 (11.0) |  |
| Craft & Related Trade Workers | 35 (4.2) | 12 (7.2) | 23 (3.4) |  |
| Elementary Occupation | 181 (21.6) | 20 (12) | 161 (24) |  |
| Legislators, Senior Officials & Managers | 48 (5.7) | 13 (7.8) | 35 (5.2) |  |
| Plant & Machine Operators and Assemblers | 16 (1.9) | 5 (3) | 11 (1.6) |  |
| Professionals | 195 (23.3) | 49 (29.5) | 146 (21.7) |  |
| Skilled Agricultural & Fishery Workers | 52 (6.2) | 12 (7.2) | 40 (6) |  |
| Skilled Workers and Shop & Market Sales Workers | 86 (10.3) | 17 (10.2) | 69 (10.3) |  |
| Technicians and Associate Professionals | 73 (8.7) | 16 (9.6) | 57 (8.5) |  |
| Unemployed | 53 (6.3) | 14 (8.4) | 39 (5.8) |  |
| Missing | 17 (2) | 0 (0) | 17 (2.5) |  |
| **Education** N(%) |  |  |  | 0.001 |
| Graduate | 317 (37.8) | 75 (45.2) | 242 (36) |  |
| High school certificate | 169 (20.2) | 23 (13.9) | 146 (21.7) |  |
| Illiterate | 15 (1.8) | 4 (2.4) | 11 (1.6) |  |
| Intermediate or diploma | 120 (14.3) | 28 (16.9) | 92 (13.7) |  |
| Middle school certificate | 108 (12.9) | 12 (7.2) | 96 (14.3) |  |
| Primary school certificate | 50 (6) | 8 (4.8) | 42 (6.2) |  |
| Profession or Honours | 46 (5.5) | 16 (9.6) | 30 (4.5) |  |
| Missing | 13 (1.6) | 0 (0) | 13 (1.9) |  |
| **Income** N(%) |  |  |  | 0.02 |
| ≤ 10,001 | 95 (11.3) | 13 (7.8) | 82 (12.2) |  |
| ≥ 199,862 | 32 (3.8) | 13 (7.8) | 19 (2.8) |  |
| 10,002–29,972 | 303 (36.2) | 57 (34.3) | 246 (36.6) |  |
| 29,973–49,961 | 152 (18.1) | 28 (16.9) | 124 (18.5) |  |
| 49,962–74,755 | 80 (9.5) | 14 (8.4) | 66 (9.8) |  |
| 74,756 –99,930 | 43 (5.1) | 6 (3.6) | 37 (5.5) |  |
| 99,931–199,861 | 34 (4.1) | 8 (4.8) | 26 (3.9) |  |
| Missing | 99 (11.8) | 27 (16.3) | 72 (10.7) |  |

**Supplementary Figure 1: Clinical Frailty Scale (from Rockwood et al. CMAJ 2005;173:489-95)**

**Supplementary Figure 2: Flow of patients in the study**


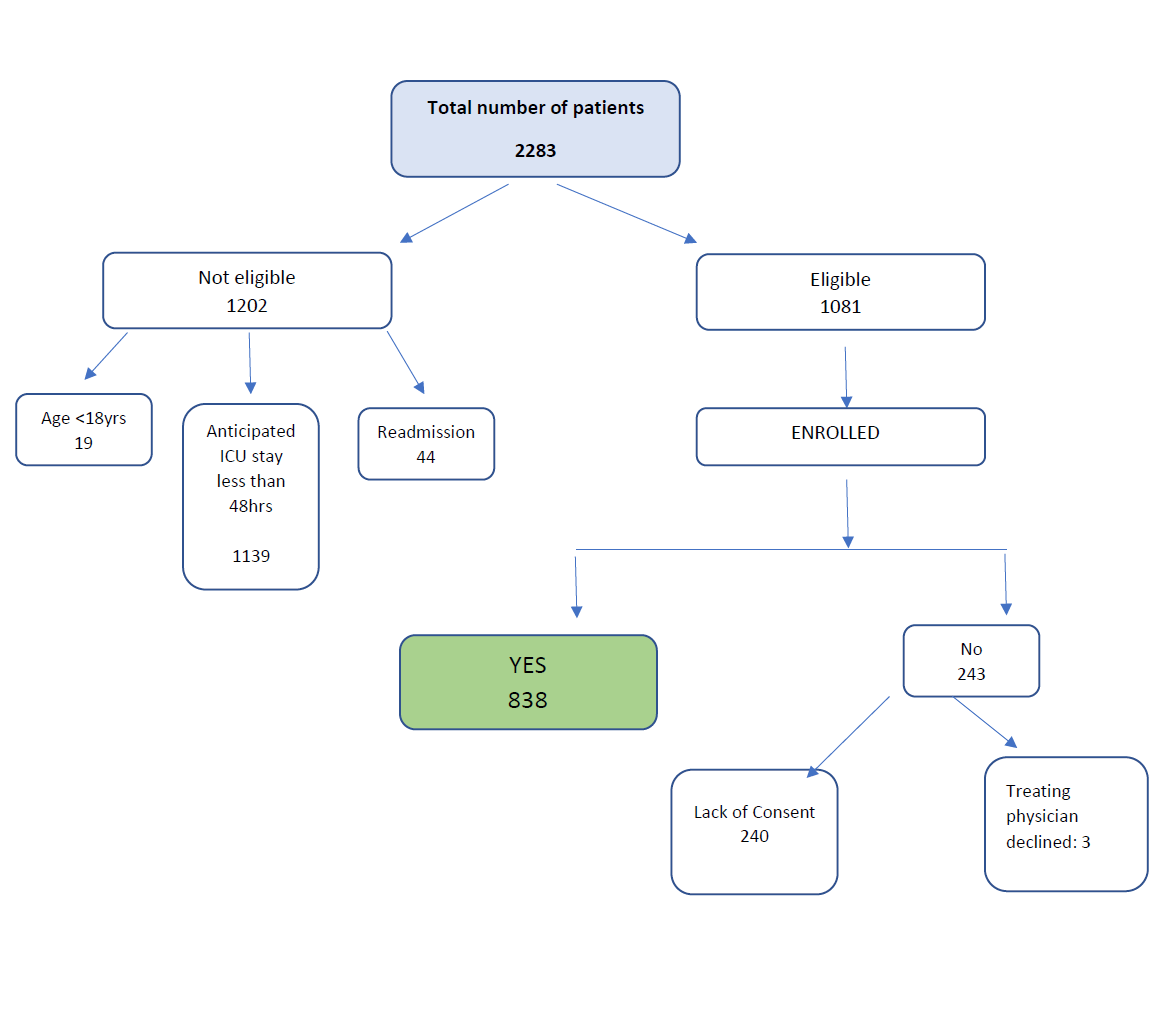


**Supplementary Figure 3: Possible mechanism explaining the protective effect of lower SES on frailty (potential collider bias)**


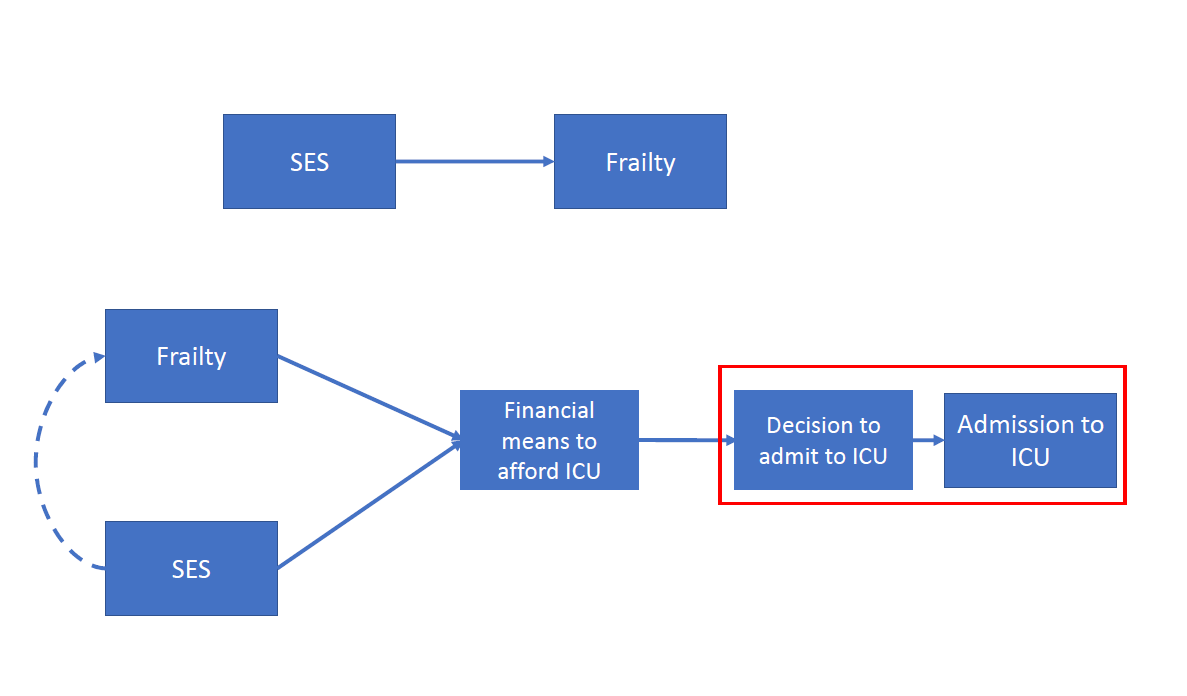


Supplementary Figure 4: Age distribution of the study population

*
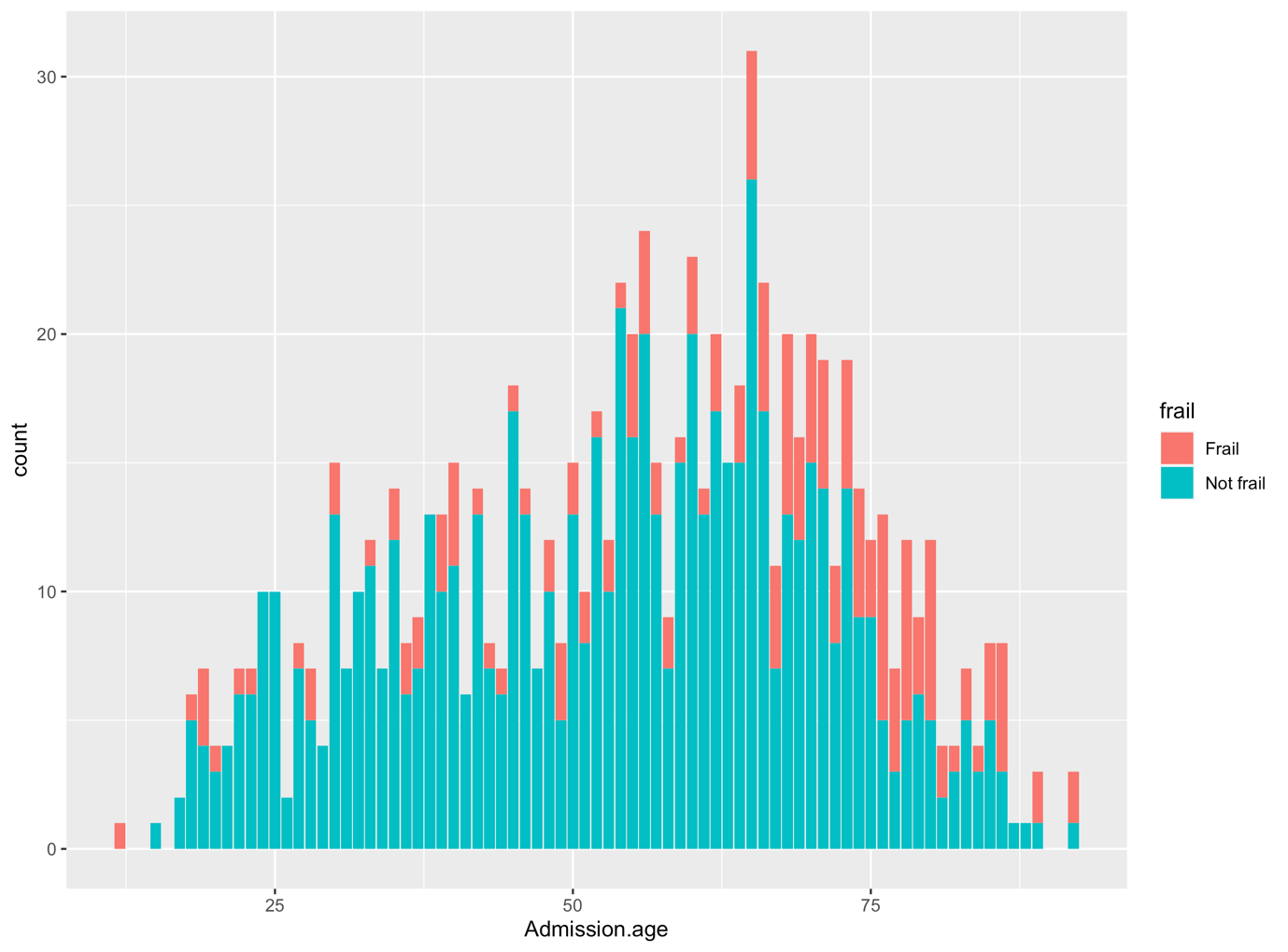
*
